# Arginase: Biological and Therapeutic Implications in Diabetes Mellitus and Its Complications

**DOI:** 10.1155/2022/2419412

**Published:** 2022-10-26

**Authors:** Yuanyuan Ren, Zhuozhuo Li, Wenqing Li, Xiaobin Fan, Feifei Han, Yaoyao Huang, Yi Yu, Lu Qian, Yuyan Xiong

**Affiliations:** ^1^Xi'an Key Laboratory of Cardiovascular and Cerebrovascular Diseases, Xi'an No.3 Hospital, The Affiliated Hospital of Northwest University, Faculty of Life Sciences and Medicine, Northwest University, Xi'an, Shaanxi, China; ^2^Key Laboratory of Resource Biology and Biotechnology in Western China, Ministry of Education, School of Medicine, Northwest University, Xi'an, Shaanxi, China; ^3^Department of Obstetrics and Gynecology, Xi'an No.3 Hospital, The Affiliated Hospital of Northwest University, Northwest University, Xi'an, Shaanxi, China; ^4^Department of Endocrinology, Xi'an No.3 Hospital, The Affiliated Hospital of Northwest University, Northwest University, Xi'an, Shaanxi, China

## Abstract

Arginase is a ubiquitous enzyme in the urea cycle (UC) that hydrolyzes L-arginine to urea and L-ornithine. Two mammalian arginase isoforms, arginase1 (ARG1) and arginase2 (ARG2), play a vital role in the regulation of *β*-cell functions, insulin resistance (IR), and vascular complications via modulating L-arginine metabolism, nitric oxide (NO) production, and inflammatory responses as well as oxidative stress. Basic and clinical studies reveal that abnormal alterations of arginase expression and activity are strongly associated with the onset and development of diabetes mellitus (DM) and its complications. As a result, targeting arginase may be a novel and promising approach for DM treatment. An increasing number of arginase inhibitors, including chemical and natural inhibitors, have been developed and shown to protect against the development of DM and its complications. In this review, we discuss the fundamental features of arginase. Next, the regulatory roles and underlying mechanisms of arginase in the pathogenesis and progression of DM and its complications are explored. Furthermore, we review the development and discuss the challenges of arginase inhibitors in treating DM and its related pathologies.

## 1. Introduction

Diabetes mellitus (DM), one of the most prevalent chronic metabolic diseases, which leads to life-threatening, disabling, and costly complications and compromises life expectancy [1]. There are two primary forms of DM: type 1 diabetes mellitus (T1DM) and type 2 diabetes mellitus (T2DM). Fundamental pathogenic differences exist in the two types of DM, of which T1DM is insulin-dependent [2]. T2DM is the most common type of diabetes, accounting for more than 90% and is characterized by insulin resistance (IR) and/or *β*-cell dysfunction [3]. Intriguingly, abnormalities in NO production, inflammatory responses, and oxidative stress have all been implicated in the development and progression of DM and its complications [4]. In the past few decades, researchers have been working to interpret the underlying mechanisms of DM pathogenesis and progression in order to seek effective and efficient therapeutic targets. Nevertheless, the exact mechanism remains elusive.

Arginase is a binuclear manganese-containing metalloenzyme that catalyzes the conversion of L-arginine to L-ornithine and urea in the last reaction of the urea cycle (UC) [5]. Available data have demonstrated the pathophysiological importance of aberrant arginase expression in hypertension [6], obesity [7], aging [8], diabetes [9], etc. Currently, arginase is being considered by the scientific community as a potential biomarker for the progression and severity of these diseases. In type 2 diabetic patients, plasma arginase activity was increased by 50% in diabetic versus control group [10]. Accumulating studies have revealed that arginase may contribute to the progression of DM and its complications, owing to its regulatory role in *β*-cell functions [11], IR [12], and vascular dysfunction [13] via mediating L-arginine metabolism, inflammatory responses, and oxidative stress. Alterations of arginase expression and activity have been confirmed in experimental and clinical investigations as a diagnostic tool for the progression of DM and its complications [9, 10, 14–17]. Therefore, arginase may represent an appealing and prospective pharmacological target for the treatment of DM and its complications.

Spurred by this context, here we conducted a thorough review of arginase's biological functions in diabetes-related pathological processes, as well as its mechanism of action in DM complications. The developmental progress and challenges of arginase inhibitors in treating DM and related complications are also highlighted. Moreover, we offer several potential approaches to tackle the issues concerning the clinical application of arginase as the diagnostic tool and therapeutic target for DM and its complications. We hope this knowledge will help us better understand the functions of arginase in DM pathogenesis and provide a reference for future clinical development of arginase in DM therapy.

## 2. The Features of Arginase and Its Roles in DM Pathology

### 2.1. Arginase Isoforms

Arginase, a ubiquitous metalloenzyme with L-arginine hydrolase activity, which has been found in bacteria, yeasts, plants, invertebrates, and vertebrates, plays a critical role in both physiological and pathological conditions [18]. In mammals, there are two distinct isoforms of arginases, arginase1 (ARG1) and arginase2 (ARG2). Despite the fact that both isoforms are present throughout the body and present similar physicochemical properties, they differ in encoding genes, expression patterns, and physiological activities as well as molecular regulation [19]. ARG1, a cytoplasmic enzyme mainly expressed in the liver and also exists in extrahepatic tissues, is located on chromosome 6q23 and encodes a 322 amino acid protein [20]. Whereas ARG2, a mitochondrial enzyme widely expressed in the kidney and some extrahepatic tissues (such as the heart, blood vessels, prostate, gastrointestinal tract, muscle, and endocrine tissues), is found on chromosome 14q24.1 and encodes a 354 amino acid protein [21]. The different biochemical environments of tissues favor the complementary roles of these two isomers in the body. ARG1 primarily functions in the UC to remove toxic ammonia and fight inflammation. ARG2 has been shown to modulate cellular L-arginine metabolism, polyamine synthesis, NO homeostasis, and proinflammation as well as oxidative stress [22].

### 2.2. L-Arginine Metabolism

**Figure 1:**
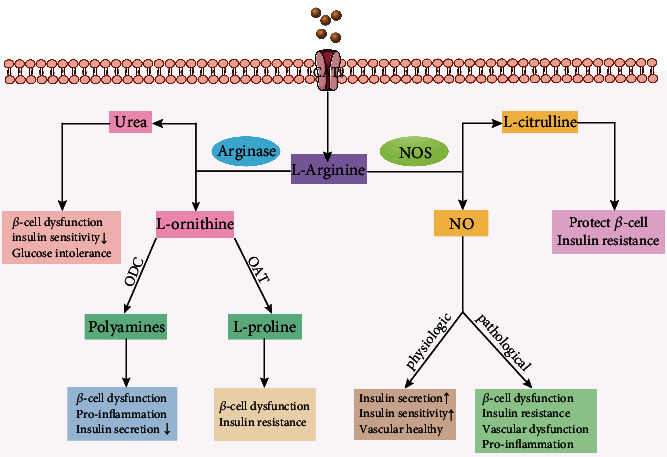
Scheme of competitive L-arginine metabolism via arginase and NOS. Arginase cleaves L-arginine to urea and L-ornithine. L-ornithine is further metabolized by ODC to synthesize polyamines, which promote *β*-cell dysfunction, insulin secretion reduction, and inflammation response, or by OAT to form L-proline, which promotes *β*-cell dysfunction and IR. Urea is implicated in *β*-cell dysfunction, insulin sensitivity reduction, and glucose intolerance. Meanwhile, NOS metabolizes arginine into L-citrulline and NO. Physiologically, NO is essential for maintaining insulin secretion, improving insulin sensitivity, and promoting vascular health. Whereas, under pathological conditions, NO has been implicated in the development of *β*-cell dysfunction, IR, vascular dysfunction, proinflammatory responses, etc. L-citrulline has also been shown to protect *β*-cell function and improve IR. ODC: ornithine decarboxylase; OAT: ornithine aminotransferase; IR: insulin resistance; NOS: nitric oxide synthase; NO: nitric oxide; CAT: cationic amino acid transporter.

Arginase catalyzes the conversion of L-arginine to L-ornithine and urea to dispose of toxic ammonia in the last step of UC. L-ornithine is further metabolized by ornithine decarboxylase (ODC) to synthesize polyamines (putrescine, spermidine, and spermine) which are involved in *β*-cell dysfunction, IR, and proinflammation, or catalyzed by ornithine aminotransferase (OAT) to form L-proline that mediates *β*-cell dysfunction and IR [23] (Figure 1). In both diabetic rats [24–27] and human patients [15, 28, 29], plasma arginine concentrations were markedly decreased, which might be positively correlated with the upregulation of arginase in DM [30]. Experimental and clinical data confirmed that L-arginine supplementation might be helpful in improving insulin secretion [31] and insulin sensitivity [32] as well as glucose tolerance [33]. Urea, another metabolite of arginase-mediated L-arginine metabolism, has been demonstrated that it is implicated in *β*-cell dysfunction, insulin sensitivity reduction, and glucose intolerance [34, 35]. A clinical study found that elevated blood urea nitrogen (BUN) levels significantly increased the risk of incident T2DM in humans [36]. Compared to healthy subjects, the level of salivary urea was elevated in diabetic patients [37]. Moreover, increased serum levels of urea were observed to be significantly associated with the severity of diabetic retinopathy (DR) [38]. In mammals, L-ornithine is a crucial precursor for polyamines and L-proline biosynthesis. Elevated ornithine was recently reported to be specifically correlated with an increased risk of T2DM [39]. In T2DM patients with dysregulated polyamine metabolism, serum putrescine and spermine levels are significantly elevated [40]. Accumulated polyamines have been shown to promote the pathogenesis of T1DM via inducing *β*-cell dysfunction and enrichment of proinflammatory immune cells [41, 42]. Furthermore, impaired glucose-stimulated insulin secretion was also observed in transgenic mice with hyperactivation of the polyamine catabolic pathway [43]. Increased polyamine synthesis exacerbates DM complications in the kidney [44] and liver [45] of diabetes animal models. L-proline can be partially synthesized from L-ornithine. It has also been discovered to be elevated in T2DM patients [46], and excessive L-proline contributes to *β*-cell dysfunction [47] and insulin resistance (IR) [48]. L-citrulline, a product of nitric oxide synthase (NOS) catalyzing L-arginine, has been shown to improve IR, which is associated with enhanced insulin sensitivity [49]. In rat *β*-cells, L-citrulline at a physiologic concentration increased glucose-stimulated insulin release [50]. Considering the essential role of arginase in the metabolism of L-arginine, abnormal arginase activity and expression are doomed to influence the progression of DM.

### 2.3. Arginase and NO Production

L-arginine also serves as a specific substrate for nitric oxide synthase (NOS), which metabolizes explicitly L-arginine to produce L-citrulline and NO [51] (Figure 1). Consequently, under conditions of excessive arginase activity, it will compete with NOS for L-arginine, eventually leading to NOS uncoupling, producing less NO and more superoxide [52, 53]. Arginase-mediated removal of L-arginine is also able to suppress inducible NOS (iNOS) expression via decreasing the translation and stability of iNOS proteins, resulting in the reduction of intracellular NO production [54]. Additionally, arginase-mediated urea production may also regulate NO generation [55].

NO, a key signaling molecule and unique gas transmitter, is associated with the development of DM due to its role in the modulation of insulin secretion and glucose homeostasis [56]. It serves as a Janus-faced molecule in DM and its complications, the effect of which is mainly dependent on its concentration and NOS isoform. At the physiological level, it is of the essence in maintaining insulin secretion, improving insulin signaling and sensitivity, increasing peripheral glucose uptake, and reducing hepatic glucose output (Figure 1). In endothelial nitric oxide synthase- (eNOS-) deficient mice, impaired NO synthesis is explicitly related to the development of IR rather than insulin-stimulated glucose uptake [57]. Whereas excessive NO (mostly iNOS derived) induced by certain pathological factors, including obesity and inflammation, results in *β*-cell dysfunction, insulin secretion impairment, and hyperglycemia as well as the development of adipose tissue IR [58–60] (Figure 1). Furthermore, aberrant NO levels in endothelial cells (ECs), vascular smooth muscle cells (VSMCs), macrophages, keratinocytes, and corpus cavernosum tissue contribute to the development of DM complications, including diabetic cardiovascular disease [61], diabetic nephropathy [62], diabetic retinopathy [63], diabetic wound-healing disorder [64], and diabetic erectile dysfunction [65]. As such, the dysregulated NO level provoked by an imbalance between arginase and NOS contributes to the pathophysiology of DM and its complications.

### 2.4. Arginase Mediates Inflammation

**Figure 2:**
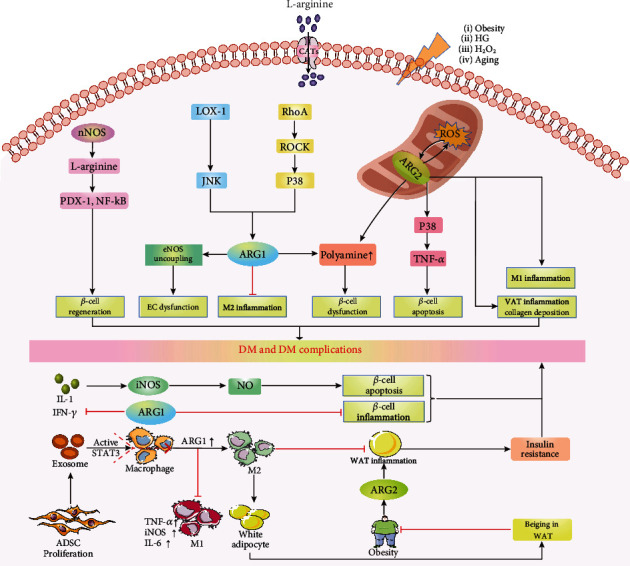
Arginase promotes DM and DM complications through multiplicate signaling pathways. Obesity/HG/oxidative species enhance ARG1 expression and exacerbate eNOS uncoupling by activating pathways of LOX-1-JNK or Rho-ROCK-P38MAPK, leading to EC dysfunction, ultimately resulting in DM complications. Upregulation of ARG1 expression in M2 macrophages inhibits inflammatory response. Furthermore, nNOS-mediated L-arginine-NO production triggers *β*-cell regeneration through activation of the transcription factors PDX-1 and NF-*κ*B. Both ARG1 and ARG2 can promote *β*-cell dysfunction via polyamine metabolism. Besides, upregulated ARG2 expression in acinar cells during aging activates p38 MAPK, which induces the release of paracrine TNF-*α*, resulting in *β*-cell apoptosis. ARG2 promotes VAT inflammation and collagen deposition, eventually leading to IR. ARG2 facilitates the inflammatory response of M1 macrophages by interacting with ROS. ADSC-derived exosomes induce anti-inflammatory M2 macrophages by transactivating ARG1, which promotes WAT beiging and metabolic homeostasis, thereby improving IR. ARG1 modulates proinflammatory cytokines- (IL-1 and IFN-*γ*) induced *β*-cells apoptosis and dysfunction via the excessive NO production from iNOS activation, ultimately accelerating the progression of DM and DM complications. HG: high glucose; ARG1: arginase 1; eNOS: endothelial nitric oxide synthase; LOX-1: lectin-like oxidized low-density lipoprotein receptor-1; JNK: c-Jun N-terminal kinase; ROCK: RhoA/rho kinase; p38MAPKs: p38 mitogen-activated protein kinases; EC: endothelial cell; DM: diabetes mellitus; nNOS: neuronal nitric oxide synthase; NO: nitric oxide; PDX-1: pancreas duodenum homeobox-1; NF-kB: nuclear factor-kB; ARG2: arginase 2; TNF-*α*: tumor necrosis factor alpha; VAT: visceral adipose tissue; IR: insulin resistance; ROS: reactive oxygen species; ADSCs: adipose-derived stem cells; WAT: white adipose tissue; IL-1: interleukin-1; IFN-*γ*: interferon-*γ*; iNOS: inducible nitric oxide synthase.

DM and its complication are inflammatory diseases [66]. Over the past few years, increasing solid evidence has demonstrated that arginase is involved in mediating pro- and anti-inflammatory responses linked to the pathology of DM and its complications. ARG1, mainly expressed in M2-like macrophages, protects inflammatory tissue from damage and clears pathogens by decreasing intracellular iNOS bioavailability of L-arginine [67]. In rat *β*-cells and RINm5F cells, inhibition of ARG1 expression resulted in aggravation of insulitis, which is an inflammatory lesion and a pathologic hallmark of T1DM [68, 69]. Transactivation of macrophage ARG1 drives an anti-inflammatory M2 phenotype, which lowers inflammation, promotes white adipose tissue (WAT) beiging, and maintains metabolic homeostasis in WAT, thereby reducing the risk of obesity-related DM [70] (Figure 2). Whereas, the elevation of ARG1 in ECs induces eNOS uncoupling that limits NO production and enhances reactive oxygen species (ROS) generation, resulting in a proinflammatory response [71] (Figure 2). In high fat-high sucrose- (HFHS-) stimulated obesity mice, endothelial-specific ARG1 knockout attenuates obesity-induced adipose tissue inflammation via maintaining endothelial NO levels [72]. By contrast, ARG2 appears to function as a proinflammatory M1-like phenotype [67]. Our previous studies in vitro and in vivo showed that targeted disruption of the ARG2 gene prevents high-fat diet- (HFD-) induced IR by suppressing the proinflammatory response of macrophage in mice [73] (Figure 2). In the aging-associated T2DM mice model, ARG2 is mainly expressed in acinar cells and upregulated with aging, which promotes tumor necrosis factor-*α* (TNF-*α*) release from pancreatic acinar cells, ultimately resulting in *β*-cell apoptosis and subsequent reduction of insulin secretion [11]. In adipose tissue and ECs, disruption of ARG2 reduces aging-related inflammation [74, 75]. In mice model, ARG2 deletion prevents HFHS-induced collagen deposition and visceral adipose tissue (VAT) inflammation, enhances adipocyte metabolism, and improves IR [76] (Figure 2). Moreover, ARG2 deficient mice have been shown to protect HFD-induced DM complication (hepatic steatosis) via inhibition of liver macrophage-mediated proinflammatory responses [77]. As L-arginine displays anti-inflammatory effects, aberrant arginase expression and activity induces the dysregulation of intracellular L-arginine, which is essential for pancreatic *β*-cell functional integrity, metabolism, and defense from an inflammatory challenge, thereby modulating insulin sensitivity and secretion [78]. These findings uncover that the two isoforms of arginase exert different functions in regulating inflammatory responses, contributing to the pathological progression and prognosis of DM and its complications.

### 2.5. Arginase and Reactive Oxygen Species (ROS)

Reactive oxygen species (ROS) is thought to be one of the culprits to the induction and progression of DM and its complications, owing to an excess of ROS that causes oxidative stress, which promotes *β*-cell dysfunction, IR, and vascular dysfunction by activating multiple cellular stress-sensitive signaling pathways [66, 79]. To date, the emerging evidence suggests that arginase regulates ROS generation upon various pathological stimuli, which further modulates the progression of DM and its complications in particular. In streptozotocin- (STZ-) induced diabetic rat model, significantly increased arginase activity and ROS levels were observed. In contrast, suppression of arginase by almond treatment remarkably ameliorated blood glucose levels and vasculogenic erectile dysfunction via the reduction of ROS production [80]. Red blood cells (RBCs) from T2DM patients display higher levels of arginase activity and ARG1 protein expression, which can induce endothelial but not smooth muscle cell dysfunction in both healthy rat aortas and human internal mammary arteries through a ROS-dependent manner [81]. Diabetic mice and retinal ECs treated with high glucose (HG) or H_2_O_2_, showed prominent increases in ROS formation and ARG1 expression and activity, which lead to ECs premature senescence [82]. Our previous study discloses that obesity-induced ARG2 upregulation enhances mitochondrial ROS production, subsequently accelerating the development of obesity-associated IR [73] (Figure 2). Urea, as a crucial metabolite of arginase-mediated L-arginine metabolism, its infusion in normal animals has been shown to induce IR and elevation of IR-related adipokines as a consequence of excessive ROS generation [83]. Additionally, arginase inhibition boosting endogenous NO production helps to dissipate ROS and promote *β*-cell survival, leading to the amelioration of insulin release [84]. However, overproduced NO may react with ROS to generate peroxynitrite, which triggers *β*-cell dysfunction and death [85], contributing to the onset of DM in nonobese diabetic (NOD) mice [86]. In this context, the delicate interaction between arginase and ROS may represent a novel mechanism of DM and its complications pathogenesis.

## 3. Roles of Arginase in the Regulation of *β*-Cell Function and IR

### 3.1. Arginase and *β*-Cell Function

Destruction or dysfunction of insulin-producing pancreatic *β*-cells persists throughout the pathological course of T1DM and T2DM. Accumulating evidence demonstrates that arginase is implicated with DM development via the mediation of *β*-cell functions [87]. Immunohistochemical analysis of mice pancreas showed that two arginase activities were indeed present in the pancreas. ARG1 but not ARG2 was detected in islets, and ARG2 was moderately expressed in acini [88]. Constitutive arginase activity and ARG1 are detected in freshly isolated rat islets of Langerhans and RINm5F cells [89]. However, compared to ARG1, ARG2 is dominantly expressed in human pancreatic islets [90]. In various models, arginase has been suggested to directly or indirectly modulate *β*-cells function through regulating inflammatory response, NO production, and L-arginine metabolism. For example, ARG1 has been shown to modulate proinflammatory cytokines- (IL-1 and IFN-*γ*) induced *β*-cells apoptosis and dysfunction via the excessive NO production from iNOS activation [69, 90, 91] (Figure 2). In our previous study, upregulated ARG2 expression in acinar cells during aging activate p38 MAPK, which induces the release of paracrine TNF-*α*, resulting in the *β*-cell apoptosis and insufficient insulin secretion, contributing to the aging-associated glucose intolerance [11] (Figure 2). Fu et al. found that in arginase-mediated ureagenesis diminishes arginine utilization for producing NO, which protects *β*-cells from inflammation and death [92]. Polyamines, the metabolite of arginase-catalyzed arginine, were found to be restricted to the insulin-producing *β*-cells; its depletion in mouse models of STZ-induced T1DM can protect islet *β*-cell from inflammation-induced dysfunction and destruction [93, 94] (Figure 2). Recently, *β*-cells regeneration is expected to offer a novel therapy for DM. In alloxan-induced diabetic rats, targeting neuronal nitric oxide synthase (nNOS) in arginine metabolic pathway ameliorates blood insulin and glucose levels in a manner of stimulating *β*-cell neogenesis via activating pancreas duodenum homeobox-1 (PDX-1) and nuclear factor-kappa-B (NF-kB) [95] (Figure 2). Besides, inhibiting polyamine biosynthesis by either *α*-difluoromethylornithine (DFMO) (NCT02384889) or imatinib (NCT01781975) could also enhance *β*-cell regeneration in the setting of DM [96]. This compelling evidence reveals the important implications of arginase in the regulation of *β*-cell mass and function.

### 3.2. Arginase and Insulin Resistance

Insulin resistance (IR), also known as damaged insulin sensitivity, is a fundamental aspect of the etiology of T2DM and is also linked to obesity [97]. Over the past decade, arginase has been verified to be implicated in the development of IR. In epididymal white adipose tissue (eWAT), abnormal ARG1 expression induced by an imbalance of M1- and M2-macrophage proportions is able to provoke adipose tissue dysfunction and obesity-related IR [58]. In HFD mice, upregulation of ARG1 reduces infiltration of macrophages in adipose tissue and facilitates polarization of macrophages to M2, thus alleviating obesity and improving insulin sensitivity [98]. Additionally, exosomes from adipose-derived stem cells (ADSCs) facilitate immune and metabolic homeostasis in WAT through the transactivation of ARG1 by exosome-carried active STAT3, thereby relieving obesity-related IR [70] (Figure 2). ARG2, also has been found to be upregulated in obesity mice, which contributes to IR via the promotion of hydrogen peroxide production and proinflammatory responses. Furthermore, ARG2-deficient mice showed lower fasting blood glucose and improved glucose tolerance and insulin sensitivity [73]. In obese Zucker rats (ZR) with IR, arginase inhibition enhances insulin sensitivity [12]. More important, in clinical practice, elevated arginase activity is detected in the plasma of T2DM patients, while IR causes a decrease in NOS activity through producing methylated arginine [10]. These studies indicate that arginase may represent a promising therapeutic target for ameliorating obesity-associated IR. Nevertheless, the underlying mechanisms of ARG1/ARG2 modulate IR still requires further investigation.

## 4. Arginase in DM Complications

**Figure 3:**
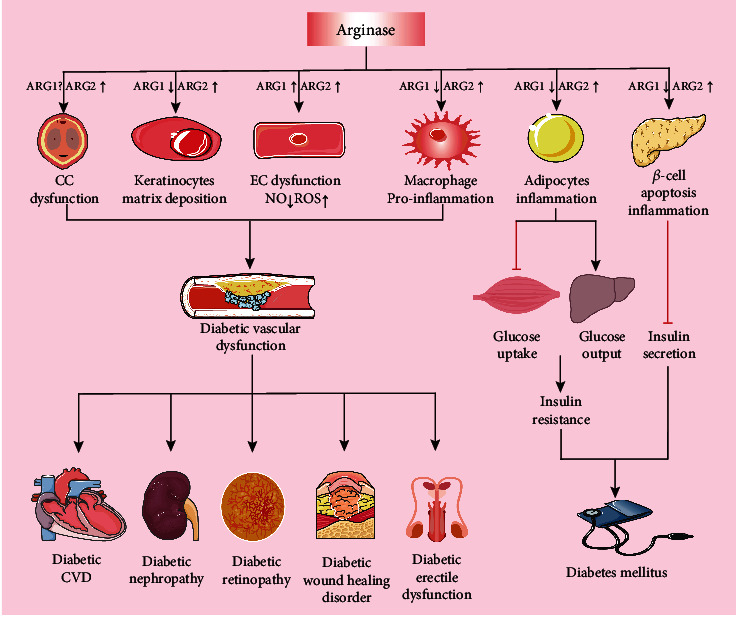
DM complications and risk factors associated with upregulation of arginase. Two mammalian arginase isoforms, ARG1 and ARG2, play a vital role in the regulation of *β*-cell functions, insulin resistance (IR), and diabetic vascular complications. Decreased ARG1 activity or expression impairs the normal function of keratinocytes, ECs, macrophages, adipocytes, and *β*-cells through enhancing proinflammatory responses, leading to DM and its complications. Whereas elevation of ARG1 in ECs induces eNOS uncoupling that limits NO production and enhances ROS generation, resulting in diabetic vascular dysfunction. Furthermore, upregulation of ARG2 activity further contributes to IR and insufficient insulin secretion through inducing adipocytes and *β*-cells dysfunction, respectively, which promotes the progression of DM. DM: diabetes mellitus; ARG1: arginase 1; ECs: endothelial cells; eNOS: endothelial nitric oxide synthase; ROS: reactive oxygen species; ARG2: arginase 2; IR: insulin resistance; CC: corpus cavernosum; CVD: cardiovascular disease.

DM, not a single disease, is also strongly associated with both microvascular and macrovascular complications, including macrovascular diseases (cardiovascular disease, CVD) and microvascular diseases (diabetic nephropathy, retinopathy, and wound-healing disorder), leading to the major cause of morbidity and mortality in individuals with DM [99]. To date, etiologies of DM vascular complications have not yet been fully elucidated. Most notably, both ARG1 and ARG2 have been identified as crucial modulators in the pathogenesis of DM complications [52, 100–102], and targeting arginase is capable of improving macrovascular and microvascular complications in DM patients [15, 16, 103, 104] (Table 1) (Figure 3).

### 4.1. Arginase and Diabetic Cardiovascular Disease

CVD increases 2-4 times in adults with DM, and the risk increases dramatically with worsening glycemic control. Increased activity and expression of arginase have been reported to exacerbate pathological diabetic CVD, such as coronary artery disease (CAD), ischemia-reperfusion (I/R) injury, and hypertension, by lowering NO generation, boosting ROS production, and proinflammation [21, 105]. Clinically, ARG1 is found in the walls of coronary arterioles in T1DM or T2DM patients but not in the nondiabetic group [15]. In the diabetes-related HG model, upregulated ARG1 induced eNOS uncoupling through the sequential activation of RhoA/Rho kinase (ROCK) and p38 mitogen-activated protein kinases (p38 MAPK) in mouse aortic and bovine aortic endothelial cells (BAECs), contributing to the development of diabetes/hyperglycemia-induced vascular endothelial dysfunction [106, 107] (Figure 2). In addition, sequential activation of low-density lipoprotein receptor-1 (LOX-1), c-Jun N-terminal kinase (JNK), and ARG1 induces ROS-dependent oxidative stress and impairs coronary arteriolar function during DM [108] (Figure 2). In STZ-induced diabetic Wistar rats, activation of p38 MAPK promotes DM-induced endothelial dysfunction via selectively upregulating the expression of ARG1 in coronary arteries and the expression of ARG2 in mesenteric arteries [109]. Our group also found that increase of ARG2 promoted eNOS uncoupling and vascular dysfunction via the activation of p38 MAPK in HFD-induced obesity mice, which could be prevented by ARG2 gene knockout [110]. ARG2 expression is significantly enhanced in the aorta and myocardium of Goto-Kakizaki (GK) rats with T2DM. Disrupting ARG2 activity by arginase inhibitor restores coronary microvascular function through a mechanism related to the increased NO availability [111]. Importantly, a clinical study showed that arginase inhibition improved isolated coronary dilation and protected against endothelial dysfunction caused by I/R in DM patients with CAD [17]. Hypertension is also commonly associated with DM. Increased vascular ARG1 expression and arginase activity have been associated with higher blood pressure in numerous experimental models of hypertension [112]. STZ-induced DM is accompanied by the elevation in systolic and diastolic blood pressure and arginase activity. In contrast, arginase inhibition mitigates DM-induced hypertension through preventing the impairment of endothelial-dependent relaxation and NO production [113]. Therefore, arginase might be considered as a novel marker for the diagnosis of DM vascular complications.

### 4.2. Arginase and Diabetic Nephropathy

Diabetic nephropathy (DN) is one of the terrifying chronic microvascular complications of DM and the leading cause of end-stage renal disease (ESRD) [114]. Inflammation and mitochondrial dysfunction have been identified as the key pathogenic factors in DN development [115]. ARG1 is reduced in STZ-administrated diabetic kidneys. Inducing ARG1 expression in renal macrophages can prevent the progression of DN via alleviating inflammation and mitochondrial dysfunction in tubular epithelial cells (TECs) [116]. Macrophage-specific deletion of ARG1 reduces macrophage infiltration but does not affect albuminuria as an early DN marker in STZ-induced DM [117]. On the contrary, after 6 and 18 weeks of STZ administration, kidney arginase activity and ARG2 exhibited significant elevation in wild-type (WT) mice, which was associated with a reduction in renal medullary blood flow and diabetic renal injury [118] (Figure 3). ARG2 expression was also increased in the renal cortex of HFD-induced obese mice. Inhibition of ARG2 was able to protect mouse kidneys from proinflammatory responses to ameliorate DN [119]. Significantly, pharmacological blockade or genetic deficiency of ARG2 reduced proteinuria levels and renal histopathological changes and lowered blood urea nitrogen and macrophage recruitment, thereby slowing down the development of DN [118, 120] (Figure 3). Further research disclosed that arginase inhibition protects renal tissue in DN via an eNOS-dependent mechanism while simultaneously having an eNOS-independent effect on renal macrophage recruitment [121]. Thus, targeting arginase, particularly ARG2, could be a new potential therapeutic intervention for DN treatment.

### 4.3. Arginase and Diabetic Retinopathy

Diabetic retinopathy (DR) is a common microvascular disorder of DM and a leading cause of blindness. The bulk of accumulating studies suggest that arginase is involved in the mediation of DR pathophysiological progression. Recently, a clinical study claims that ARG1 rs2781666 single nucleotide polymorphism (SNP) is substantially linked to DR susceptibility in T2DM patients [122]. Retinal ECs senescence under HG condition is the main pathomechanism of DR. Retinal ECs treated with HG or H_2_O_2_ showed prominent increases in arginase expression and activity, which evoked retinal ECs senescence through a mechanism related to NADPH oxidase-2- (NOX2-) generated ROS and decrease in NO bioavailability, hastening the onset of DR [82]. In a mice model, STZ-induced DM promoting the increase in ARG1 expression accelerated retinal ECs senescence, which could be prevented by ARG1 gene deletion or pharmacological inhibition [123] (Figure 3). Elms et al. also found that the diabetes-induced vascular dysfunction was markedly attenuated in mice with heterogeneous ARG1 gene deletion (ARG1^+/-^) and in mice treated with arginase inhibitors [124]. Retinal ARG2 was similarly upregulated in HFHS diet-induced retinopathy mice model, and depletion of ARG2 was protected against the western diet-induced retinopathy via the suppression of retinal oxidative stress and inflammation [125]. Researchers studying DR patients' metabolomics found that arginine and proline dysregulated metabolism was associated with proliferative diabetic retinopathy (PDR) [126, 127]. Besides, spermine, as an arginase-modulated metabolite, is dramatically elevated in vitreous samples from patients with PDR [128]. Studies by Narayanan et al. and Liu et al. disclosed that diabetes-induced upregulation of spermine oxidase (SMOX) leads to the oxidation of spermine to spermidine, resulting in the increase in reactive aldehydes and H_2_O_2_, which are further converted to acrolein, resulting in retinal neuronal damage and dysfunction [129, 130]. Thus, arginase mediated the metabolism of arginine and proline, and polyamine metabolism might also contribute to the pathogenesis of DR. However, the underlying mechanism that arginase-related metabolites regulate DR development still requires further investigations.

### 4.4. Arginase and Diabetic Wound-Healing Disorder

Diabetic wound healing disorder, e.g., diabetic foot ulcer, is a severe complication of DM with significant morbidity and mortality, as wound healing or skin repair impairment occurs at the diabetic wound sites [131]. Arginase is expressed in a variety of wound-healing cell types, including epithelial cells, fibroblasts, polymorphonuclear cells, and macrophages [132, 133]. Convincing clinical studies showed the considerable elevation of arginase activity and protein expression in diabetic ulcers, which influences the characteristic callus formation around these ulcers [134] (Figure 3). In db/db and ob/ob diabetic mice with severe wound healing disorders, both ARG1 and ARG2 isoforms mRNA expression and arginase activity were strongly upregulated upon injury, which paralleled the expressional and activity kinetics of the iNOS. Conversely, leptin administration reduced the overall arginase activity in healing wounds, which causes a readjustment of arginases and iNOS at the wound site, improving healing [133]. After surgery, wound closure is accelerated by inhibiting arginase activity using an arginase inhibitor via hastening re-epithelialization and localization of myofibroblasts beneath the wound epithelium [135]. Notably, subcutaneous injection of arginine into the foot ulcer of diabetic patients improved local blood circulation and promoted wound healing by increasing NO-dependent blood flow and nutrient supply [136]. These studies substantially support the notion that arginase plays a vital role in regulating DM-associated wound healing through the regulation of NO production, inflammatory responses, or L-arginine metabolism.

### 4.5. Arginase and Diabetic Erectile Dysfunction

Erectile dysfunction (ED), another complication of diabetic vascular dysfunction, has a three-fold increased risk in people with diabetes compared to healthy men [137]. Both ARG1 and ARG2 are expressed in the corpus cavernosum (CC); their expression and arginase activities appear to be dysregulated in CC of diabetic individuals with ED [138–140]. Increased ARG2 but not ARG1 expression in DM patients' CC tissue, along with decreased NO generation and CC relaxation, has been found to contribute to ED [138] (Figure 3). In the animal model, the CC of WT diabetic mice displayed the enhanced arginase activity and ARG2 protein expression and the reduced phospho-eNOS at Ser-1177, while deletion of the ARG2 gene or pharmacological inhibition of arginase dramatically improved the nitrergic and endothelium-dependent relaxation in CC of diabetic mice [139]. Mechanistically, increased arginase activity caused the reduction in NO production in the cavernous tissue of DM, leading to the impairment of endothelial function and nitrogen function [141]. Additionally, activated RhoA/Rho kinase (ROCK) mediates diabetes-induced elevation of arginase expression and activity, which contributes to impaired CC relaxation probably through the activation of p38 MAPK [140]. Undeniably, targeting arginase, particularly ARG2, may represent a new approach to preventing diabetic ED [142].

## 5. Arginase Inhibitors for DM and Its Complications Therapy

Arginase inhibitors mainly comprise chemical and natural compounds. Their effects have been evaluated in DM and its complications, among which chemical arginine inhibitors include N-omega-hydroxy-L-arginine (NOHA) and its analog, 2(S)-amino-6-boronohexanoic acid (ABH), S-(2-boronethyl)-l-cysteine (BEC), and *α*-difluoromethylornithine (DFMO), of which natural arginine inhibitors comprise amino acids, polyphenolic compounds, and traditional Chinese medicine (TCM) herbs (Table 2).

### 5.1. Chemical Arginine Inhibitors

NOHA and nor-NOHA, hydroxy derivatives of arginine, are both reversible and competitive inhibitors of arginase. NOHA, a transition intermediate of NO from arginine catalyzed by NOS, is a competitive inhibitor with *K*_d_ 3.6 *μ*M (pH 8.5) for human ARG1 [143] and with *K*_i_ 1.6 *μ*M (pH 7.5) for human ARG2 [144]. In diabetic patients, arginase suppression with NOHA markedly improved coronary endothelium-dependent vasodilation [15]. nor-NOHA, a derivate of NOHA, with a longer half-life and higher affinity for arginase [145], binds to human ARG1 with *K*_d_ 0.517 *μ*M (pH 8.5) [143] and inhibits human ARG2 with *K*_i_ 51 nM (pH 7.5) [144].

In the obese Zucker rats (ZR) model, arginase inhibition by nor-NOHA ameliorates obese-induced IR and prevents the development of hypertension, while L-arginine administration only attenuates hypertension [12]. Administration of nor-NOHA in RBCs from T2DM patients has been shown to reduce ROS generation and cardiac injury postischemia-reperfusion in db/db mice [146]. Treatment with nor-NOHA for 24 days, the citrulline-NO pathway was upregulated, while the incidence of autoimmune diabetes was reduced in elderly diabetic female NOD mice [147]. Similarly, nor-NOHA administration protects I/R-induced cardiac impairment in T1DM [148]. In the registered clinical trial (NCT02009527), nor-NOHA administration suppresses the elevated arginase activity in coronary artery disease (CAD) patients with T2DM remarkably improved endothelial function following I/R, and no side effects were reported [17]. DM impairs endothelium-dependent dilation of retinal arterioles, while nor-NOHA significantly improves endothelial function of retinal arterioles in the STZ-induced diabetes pig model [149].

ABH and BEC, two boronic acid analogs, are highly selective and competitive arginase inhibitors that bind to the active manganese cluster site of arginase [150], with *K*_i_ value of 0.11 mM and 0.4~0.6 mM, respectively [151]. ABH inhibits human ARG1 with IC50 of 1.54 *μ*M and 2.55 *μ*M on human ARG2 [152]. BEC binds to human ARG1 with *K*_d_ of 270 nM and *K*_i_ of 30 nM for human ARG2 [153, 154]. In T2DM patients, plasma arginase activity is significantly elevated and accompanied by reduced NO production and impaired vasorelaxation, while inhibition of arginase by ABH (100 *μ*mol/L) restored these alternations to normal [155]. Administration with ABH for 18 hours prevented endothelium-dependent relaxation (EDR) injury induced by T2DM erythrocytes in rat aorta [81]. Inhibition of ARG1 with ABH therapy avoided the decline of NO and significantly reduced the incidence of diabetes and obesity-induced bone complications [156]. In the diabetic mice model, BEC treatment markedly improved endothelium-dependent vasorelaxation of the aortas [157]. Of note, BEC administration can also prevent the progression of established DN through the eNOS-dependent mechanism [118, 120, 121]. For DR, ABH and BEC have been shown in vivo and in vitro studies to reduce oxidative stress and alleviate diabetes-induced retinal blood flow impairment [124]. Besides, BEC was able to improve cavernosal relaxation in STZ-diabetic mice [158].

DFMO, an irreversible mixed inhibitor of arginase and ODC, has an inhibitory effect on arginase (*K*_i_ of 3.9 ± 1.0 mM on HT-29 homogenate arginase) [159]. Its administration significantly improved the diabetic endothelial-dependent vasodilatory response via inhibiting arginase activity [52].

### 5.2. Natural Arginine Inhibitors

A portion of the natural amino acids have been discovered to effectively decrease arginase activity, preventing diabetes and its complications. L-citrulline, an amino acid present in watermelon [160], has been reported to be an allosteric inhibitor of bovine liver arginase with 53% inhibition at 20 mM [161]. L-citrulline administrated hepatoma H4IIE cells, and SHRSP.Z-Leprfa/IzmDmcr rats presented the improvement in insulin sensitivity [49]. Clinically, T2DM patients taking L-citrulline supplements (2000 mg/day) for one month have been shown to decrease arginase activity by 21% and meanwhile improve glycated hemoglobin (HbA1c) levels and plasma NO production (NCT03358264) [162]. In vitro and vivo studies, treatment with the arginase inhibitor L-citrulline (1 mmol/L) effectively blocked the HG-induced increase in arginase activity and superoxide formation in bovine coronary endothelial cells (BCECs) and reversed diabetes-impaired coronary endothelial cell-dependent vasorelaxation in the STZ-induced diabetic rats model [52]. Excitingly, clinical studies have confirmed that amino acids are capable of producing the minimum side effects compared to other medical treatments [163]. L-norvaline is also a powerful arginase inhibitor and a unique compound with a wide range of biological characteristics [164]. Because of its structural similarities to ornithine, it inhibits NO synthesis via a negative feedback mechanism and significantly increases NO production rate [165]. In HFD/STZ-induced diabetic mice, L-norvaline treatment reduced fasting blood glucose levels by 27.1% when compared with untreated HFD/STZ mice [166]. In fructose-induced metabolic syndrome, L-norvaline administration reduced hyperinsulinaemia and hypertriglyceridaemia without affecting hyperuricaemia or hypercholesterolaemia associated with metabolic syndrome [167]. Recently, it also has been reported to improve vascular function in diabetics by decreasing arginase activity in cavernous tissue and raising NO levels [168]. L-norvaline has minimal side effects, but because of its high water solubility and high half-maximal inhibitory concentration (IC50 of 5.6 mM on rat arginase), its application in blocking the arginase pathway is still unsatisfactory [169]. High water solubility can lead to burst or uncontrolled release, while high IC50 requires high drug loading content of L-norvaline to satisfy high dosage.

Plant-derived molecules that inhibit arginase activity have also been extensively investigated. Quercetin, a bioactive plant flavonol compound, exhibits a competitive arginase inhibitory activity and inhibits Leishmania arginase with IC50 of 3.8 *μ*M [170]. In cultured skeletal muscle cells, it stimulated glucose uptake through an insulin-independent mechanism involving the activation of adenosine monophosphate-activated protein kinase (AMPK) signaling pathway [171], which is consistent with our previous study that overexpressed ARG2 inhibited the AMPK phosphorylation in ECs [172]. Treatment with a nanoformulation of quercetin for 21 days alleviated DR in zebrafish by reducing arginase activity [173]. *Moringa oleifera*, an important natural source of phenolic compounds that can inhibit rat arginase with IC50 of 159.59 *μ*g/mL [174], is an effective dietary food for the prevention and treatment of obesity and T2DM [175]. Supplementation of 5% *Moringa* in a very high-fat diet (VHFD) fed C57BL/6L mice significantly improved glucose tolerance and insulin sensitivity compared to VHFD-fed mice [175]. In addition, treatments of diabetic rats with *Moringa oleifera* had beneficial effects on the management of ED caused by DM [176]. Mechanistically, *Moringa oleifera* inhibiting arginase activity promotes the production of NO in penile tissue. Moreover, the clinical trials evaluating the effects of *Moringa oleifera* in patients with T2DM are undergoing [177–179].

With a history of over 2000 years, traditional Chinese medicine (TCM) has developed into a unique system for treating various diseases; TCM herbs show protective effects against DM and its complications by modulating arginase expression and activity. These herbs contain multiple biological molecules, which interact with each other and produce synergistic effects that strengthen therapeutic efficacy and lower the toxicity of individual herbs [180]. Semen cuscutae (SC), a well-known Chinese medicine extracted from the mature dried seeds of *Cuscuta chinensis* Lam, owns various biological properties, including antioxidant and anti-inflammation [181]. In HFD-induced obese mice, SC treatment remarkably inhibits HFD-induced increases in arginase activity and weights of liver and visceral fat tissue in a dose-dependent manner to reduce hepatic lipid metabolism and systemic adiposity via the suppression of hepatic arginase [182]. HuangqiGuizhiWuwu Decoction (HGWWD), commonly used for the treatment of diverse cardiovascular and cerebrovascular diseases in mice, was reported to lessen STZ-induced impairment of velocity and pulsatility of left femoral arteries; aortic pulse wave velocity and vascular relaxation enhance NO production in the aorta and plasma, as well as blunt endothelial arginase activity and aortic ARG1 expression [183]. In the type 1 DN mice model, Xiao-Shen-Formula (XSF) treatment improved STZ-induced renal hyper-filtration, glomerulosclerosis, and renal microvascular remodeling and prevented the increased of oxidative stress and inflammatory cytokines releases by ablating the increased levels of ARG2 protein and arginase activity, which was comparable to that of ABH treatment alone [184].

## 6. Concluding Remarks

Overall, dysregulated arginase expression and activity play a critical role in the onset and development of DM and its complications via the modulation of insulin release, IR, L-arginine metabolism, and oxidative stress as well as immune response. Therefore, monitoring the alterations of arginase activity and expression and targeting arginase offer a promising approach to diagnosing and treating DM and its complications. Nevertheless, there are still some limitations and challenges waiting for the translation of preclinical findings into therapeutic applications.

First of all, substantial clinical and experimental studies suggest that arginase could be a biomarker and diagnostic parameter for DM and its complications. However, there is no clinical definition standard of arginase activity or ARG1/2 expression levels in blood or tissues for diagnosing DM and its complications. For this purpose, it is feasible to build an artificial intelligence- (AI-) based prediction model through the deep learning of clinical data of patients with DM or DM complications, including the arginase activity values, expression levels, and patient information, to evaluate the potential risk of DM and its complications quickly. Secondly, arginase activity is indispensable for normal cellular physiological function since ARG1 exerts as the final enzyme of UC to detoxify ammonia and ARG2 is required for urine concentration in the kidney and smooth muscle cell proliferation [185, 186]. Concerning safety considerations, to lower the toxicity of arginase inhibitors, it is necessary to take into account the inhibition potency of inhibitors on ARG1 and ARG2 and which isoform of arginase dominantly contributes to the pathogenesis of DM and its complications in different individuals. Thirdly, as the distinct roles of ARG1 and ARG2 in the pathogenesis of DM and its complications, developing isoform-specific arginase inhibitors is a novel strategy to improve the therapeutic efficacy. In contrast, the high homology of the active enzymatic sites between human ARG1 and ARG2 frustrates this progress. Presently, high-resolution crystallographic structures of the enzyme, molecular and computational modeling have provided a possible route to developing hyperactive arginase inhibitors with specific properties [112]. Finally, due to the molecular diversity and low toxicity of nature arginase inhibitors, their extraction from natural medicinal plants or TCM herbs appears to be a promising approach, which not only provides new structures references for designing pharmaceutical arginase inhibitors but may also allow dietary therapy to treat DM and its complications.

## Figures and Tables

**Table 1:** The dysregulated expression of arginase in DM complications.

DM complications	Location	Arginase expression	Species/region	Effect of arginase	Inducer or activator	Refs.
Diabetic cardiovascular disease	EC	↑ARG1	Human coronary arterioles	Reduced NO production and diminished vasodilation	Increased in DM	[15]
			Mouse aorta and BAEC	Promote vascular endothelial dysfunction	Activated by RhoA/ROC, ERK1/2, and p38 MAPK	[106]
			HUVEC	Reduced eNOS activity and induced endothelial dysfunction	Activated by p38 MAPK pathway	[107]
			Pig coronary arterioles	Induced ROS-dependent oxidative stress and impaired coronary arteriolar function	Activated by LOX-1 and JNK	[108]
			Male Wistar rats coronary arteries	Promote diabetes-induced endothelial dysfunction	Activated by p38 MAPK pathway	[109]
		↑ARG2	Male Wistar rats mesenteric arteries	Reduced expression of eNOS and impaired endothelium-dependent relaxations	Activated by p38 MAPK pathway	[109]
			Mice aorta	Induced eNOS-uncoupling and vascular dysfunction	Increased in obesity	[110]
			Rat aorta and myocardium	Reduced NO availability and impaired coronary artery microvascular function	Increased in T2DM	[111]
	RBC	↑ARG1	Rat aortas and human internal mammary arteries (IMA)	Induced endothelial dysfunction	Activated by ROS	[81]
	VSMC	↑ARG1	Human coronary arterioles and internal mammary artery	Reduced NO availability and impaired endothelial dysfunction	Increased in T2DM	[16]

Diabetic nephropathy	Macrophages	↓ARG1	Mouse renal tissues	Promote inflammation and mitochondrial dysfunction	_	[116, 117]
	Renal cortex	↑ARG2	Mouse mesangial	Promote inflammation	Increased in obese	[119]
	Kidney tissue	↑ARG2	Mice kidney	Increased blood urea nitrogen and macrophage recruitment	_	[118]

Diabetic retinopathy	Peripheral blood leukocytes	ARG1	Patients with DM	Increased susceptibility to diabetic retinopathy	ARG1 rs2781666 single nucleotide polymorphism (SNP)	[122]
	Retinal EC	↑ARG1	Mice retinal vessels	Accelerated retinal ECs senescence	Increased in DM	[123]
	Central retinal artery	↑ARG1	Rat CRA	Impaired endothelial-dependent vasodilation responses	_	[124]
	Retinal EC	↑ARG2	BREC	Increased retinal oxidative stress and inflammation	Increased in HFHS-diet	[125]

Diabetic ulcer	Epidermal keratinocytes	↓ARG1 ↑ARG2	Mice wound tissue	Induced wound healing impairment	Increased in DM	[133]

Diabetic erectile dysfunction	Cavernosal tissue	↑ARG2	Human cavernosal tissue	Decreased NO generation and CC relaxation	_	[138]
			Mice cavernosal tissue	Decreased CC relaxation	Activated by ERK pathway	[158]

**Table 2:** Interventional studies with arginase inhibitors in DM and DM complications.

Arginase inhibitors	Chemical class	Dose range	Models	Effects	Refs.
*Chemical arginase inhibitors*					
NOHA	N*ω*-OH-based arginine analog	10 *μ*mol/L, 30 min (ex vivo)	Patients with DM	↑Restoration of endothelium-dependent agonist-induced dilation in coronary arterioles of patients with DM	[15]
nor-NOHA		25 mg/kg/day 4 weeks (Ip)	Zucker rats with obese	↓Prevention in the development of hypertension ↓Reduction in body weight and insulin resistance	[12]
		1 and 3 mmol/L, 20 min (ex vivo)	RBCs from diabetes mice	↑Improvement in postischemic-myocardial function	[153]
		30 mg/kg, 24 days (Ip)	Female mouse with T1DM	↓Reduction in the incidence of autoimmune diabetes	[154]
		0.1 mg/min, 20 min (Ia)	Male patients with CAD or CAD+T2DM	↑Improvement in endothelial function following ischemia-reperfusion	[17]
		0.1 mmol/L, 90 minutes intraluminal	Pigs with DM	↑Improvement in endothelial function of retinal arterioles	[149]
		100 mg/kg, 15 min (Iv)	Rat with T2DM	↑Improvement in myocardial microvascular function	[111]
		1 mL/min, 2 h (Ia)	Patients with type 2 diabetes+CAD	↑Improvement in endothelial function irrespective of glucose-lowering regimen	[16]
		0.1 mg/min, 2 h (Ia)	Patients with T2DM and microvascular dysfunction	↑Improvement in endothelium-dependent microvascular dilatation	[104]
ABH	Boronic acid-based arginine analog	8 mg/kg, 5 days (Sc)	Mouse with T1DM	↑ Improvement in retinal endothelial function	[124]
		10 mg/kg/day, 1 month (Po)	Mice with obesity and T2DM	↓Prevention in diabetic bone complications	[156]
BEC		50 *μ*mol/L, 30 minutes (ex vivo)	Mouse with T1DM	↑Improvement in endothelial function	[139]
		2.3 mg/kg/day， 6 weeks~12 weeks (Sc)	Mice with T1DM	↑Protection of kidney tissue by eNOS-dependent ↑Recruitment in kidney macrophage by eNOS-independent	[120, 121]
		100 *μ*mol/L, 45 min (ex vivo)	Mouse and rat with DM	↑Improvement in retinal vascular endothelial function	[124]
		10^−4^ mol/L (ex vivo)	Mice with DM	↑Improvement in cavernosal relaxation	[158]
DFMO	L-ornithine analog	50 *μ*mol/L, 1 h (ex vivo)	Rat with T1DM	↑Improvement in endothelial function	[52]

*Natural arginase inhibitors*					
L-citrulline	—	250 *μ*mol/L, 1 h (ex vivo), 2 g/kg/day, 8 weeks (Po)	H4IIE cell and SHRSP.Z-Leprfa/IzmDmcr rats	↑Improvement in insulin sensitivity	[49]
		2000 mg/day day, 1 month (Po)	Patients with T2DM	↑Improvement in H1Ac levels	[162]
		1 mmol/L (ex vivo)	Rat with T1DM	↑Improvement in endothelial function	[52]
		1 mmol/L， 1 h (ex vivo)	Rats with T1DM	↓Reduction in hypertension	[113]
L-norvaline	L-valine analog	20 mg/kg/day, continuing every third day for five weeks (Ip)	Mice with HFD/DM	↓Reduction in blood glucose levels	[166]
		10 mg/kg, 30 days (Ip)	Adult male rats with DM	↑Improvement in the diabetic sexual impairment	[168]
Quercetin	Polyphenolic compounds	5 and 10 mg/kg, 21 days (Ip)	Zebrafish with type 1 DR	↑Remission in diabetic retinopathy	[173]
*Moringa oleifera*		5% of *Moringa* concentrate (MC), 12 weeks	Mice with VHFD	↑Improvement in glucose tolerance and insulin sensitivity	[175]
		4% of *Moringa oleifera*, 14 days	Male rats with DM	↑Improvement in diabetic-induced ED	[176]
Semen cuscutae	Traditional Chinese medicine	0.5~10 *μ*g/mL (ex vivo)	Rats with HFD	↓Reduction in hepatic lipid metabolism and systemic adiposity	[182]
HGWWD		60 g/kg/d, 2 weeks (gavage)	Mice with T1DM	↑Improvement in vascular dysfunction	[183]
XSF		3 g/kg/d， 6 weeks (gavage)	Mice with T1DM	↓Prevention in diabetic kidney damage	[184]

Ia: intra-arterial; Ic: Intracoronar; Iv: intravenous; Ip: intraperitoneal; Sc: subcutaneous; Po: peros.

## Data Availability

The datasets used and/or analyzed during the current study are available from the corresponding author on reasonable request.

## Abbreviations

UC: Urea cycle
ARG1: Arginase1
ARG2: Arginase2
IR: Insulin resistance
NO: Nitric oxide
DM: Diabetes mellitus
T1DM: Type 1 diabetes mellitus
T2DM: Type 2 diabetes mellitus
ODC: Ornithine decarboxylase
OAT: Ornithine aminotransferase
BUN: Blood urea nitrogen
DR: Diabetic retinopathy
NOS: Nitric oxide synthase
iNOS: Inducible nitric oxide synthase
eNOS: Endothelial nitric oxide synthase
ECs: Endothelial cells
VSMCs: Vascular smooth muscle cells
WAT: White adipose tissue
ROS: Reactive oxygen species
HFHS: High fat-high sucrose
HFD: High fat diet
TNF-*α*: Tumor necrosis factor-*α*
VAT: Visceral adipose tissue
STZ: Streptozotocin
RBCs: Red blood cells
HG: High glucose
NOD: Nonobese diabetic
nNOS: Neuronal nitric oxide synthase
PDX-1: Pancreas duodenum homeobox-1
NF-kB: Nuclear factor-kappa-B
DFMO: *α*-Difluoromethylornithine
eWAT: Epididymal white adipose tissue
ADSCs: Adipose-derived stem cells
ZR: Zucker rats
CVD: Cardiovascular disease
CAD: Coronary artery disease
I/R: Ischemia-reperfusion
ROCK: RhoA/Rho kinase
p38 MAPK: p38 Mitogen-activate protein kinases
BAECs: Bovine aortic endothelial cells
LOX-1: Lipoprotein receptor-1
JNK: c-Jun N-terminal kinase
DN: Diabetic nephropathy
ESRD: End-stage renal disease
TECs: Tubular epithelial cells
WT: Wild-type
DR: Diabetic retinopathy
SNP: Single nucleotide polymorphism
NOX2: NADPH oxidase-2
PDR: Proliferative diabetic retinopathy
SMOX: Spermine oxidase
ED: Erectile dysfunction
CC: Corpus cavernosum
NOHA: N-omega-hydroxy-L-arginine
nor-NOHA: N-omega-hydroxy-nor-L-arginine
ABH: 2(S)-amino-6-boronohexanoic acid
BEC: S-(2-boronethyl)-l-cysteine
DFMO: Difluoromethylornithine
TCM: Traditional Chinese medicine
EDR: Endothelium dependent relaxation
BCECs: Bovine coronary endothelial cells
AMPK: Adenosine monophosphate-activated protein kinase
VHFD: Very high fat diet
SC: Semen cuscutae
XSF: Xiao-Shen-Formula
HGWWD: HuangqiGuizhiWuwu Decoction
AI: Artificial intelligence
CAT: Cationic amino acid transporter
IL-1: Interleukin-1
IFN-*γ*: Interferon-*γ*.
